# Implementation of the Tier 1 Program of the Project P.A.T.H.S.: Interim Evaluation Findings

**DOI:** 10.1100/tsw.2006.356

**Published:** 2006-11-16

**Authors:** Daniel T.L. Shek, Rachel C.F. Sun

**Affiliations:** ^1^ Quality of Life Centre, Hong Kong Institute of Asia-Pacific Studies, The Chinese University of Hong Kong, Hong Kong; ^2^ Social Welfare Practice and Research Centre, The Chinese University of Hong Kong, Hong Kong

**Keywords:** positive youth development, evaluation, Hong Kong, process evaluation

## Abstract

To understand the implementation quality of the Tier 1 Program of the Project P.A.T.H.S., 25 schools and three school social service units were randomly selected to participate in telephone interviews regarding the quality of the implementation process of the Tier 1 Program of the P.A.T.H.S. Project. In the telephone interviews, the participants described the responses of the students and the workers to the program, the perceived benefits of the program, their assessment of the positive and negative features of the program, as well as difficulties involved in the implementation process. Results showed that most workers perceived that the students had positive responses to the program and half of the workers had positive experiences about the program, although negative comments on the program design and difficulties in the implementation were also recorded. Nearly all workers (97.1%) regarded the program to be beneficial to the students and most of them (78.6%) had positive global evaluation of the project. In short, while the program implementers expressed concerns about the program design and the implementation process, they generally regarded the program as helpful to the students and they had positive global evaluation of the program.

### INTRODUCTION

There are many researchers who argue that the development of developmental assets in adolescents is helpful to their development[1,2]. In addition, there are views that emphasize the importance of holistic development in adolescents[3]. With reference to the Chinese culture, there is a strong emphasis on academic excellence in adolescents and the importance of holistic adolescent development is not taken seriously by Chinese parents[4]. Furthermore, there are research findings showing that adolescents in Hong Kong faced high levels of stress in different psychosocial domains[5]. Obviously, how to promote holistic development in Chinese adolescents and help them to cope with life stresses are important issues to be considered.

To promote holistic adolescent development, The Hong Kong Jockey Club Charities Trust has approved HK$400 million for a positive youth development program entitled “P.A.T.H.S. to Adulthood:

A Jockey Club Youth Enhancement Scheme". The word "P.A.T.H.S." denotes **P**ositive **A**dolescent **T**raining through **H**olistic **S**ocial Programmes. In the Tier 1 Program of the project, 15 positive youth development constructs identified from the existing successful positive youth development programs[6] are covered in the developed curriculum. To enable colleagues in the field to get familiar with the program, 52 schools were included in the Experimental Implementation Phase of the project in the academic year of 2005–06.

Obviously, it is important to ask whether the program objectives were actually achieved. To answer this question, several evaluation mechanisms were carried out to evaluate the project. First, objective outcome evaluation data utilizing a one-group pretest-posttest design was used[7]. Second, qualitative evaluation data were collected based on focus group interviews[8]. Third, process evaluation data based on systematic observations of the program implementation were collected[9]. Finally, the workers and participants were invited to complete subjective outcome evaluation forms at the end of the program using Form A and Form B developed by the researchers.

In view of the pioneering nature of the project, it is argued that process evaluation is of great importance. Hence, besides process evaluation in terms of systematic observations of the implementation of the program[9], an interim evaluation was carried out during the program implementation process to gain more understanding of the reactions of the participants and workers to the program. According to Meyer et al.[10], the development of a feedback loop from the participants and workers regarding the program implementation is important for program refinement. In this study, through process evaluation via telephone interviews, information in the following areas was collected: (1) workers' perceptions of the responses of the participants to the program, (2) experiences of the workers delivering the program, (3) perceived helpfulness of the program, (4) positive aspects of the program, (5) aspects of the program that require improvement, (6) difficulties encountered during program implementation, and (7) overall evaluation of the program. In this study, the general principles of qualitative research (e.g., use of open-ended questions without preset answers for some of the questions, consciousness of how biases might influence data interpretations, encouragement of the informants to freely narrate their views) were maintained.

### METHODS

#### Participants

In the Experimental Implementation Phase in 2005–06, 52 schools joined the project. In each school, the school social work service was operated by a Government organization (NGO). Among these schools, 29 adopted the 20h full program and 23 adopted the 10h core program. Among these participating schools, 15 schools joining the full program (i.e., 20h program), 10 schools joining the core program (i.e., 10h program), and three NGOs providing school social work services were randomly selected to join this study. For each selected school or NGO providing school social work services, the relevant contact persons were invited to participate in telephone interviews on a voluntary basis. The participants included 25 teachers and three social workers, involving 27 schools joining the project. The number of schools that participated in this research can be regarded as respectable as more than half of the participating schools of the project joined the interviews. Moreover, because the schools were randomly selected, the generalizability of the findings can be enhanced. These justifications satisfy Principle 2 in the implementation of qualitative evaluation research proposed by Shek et al.[11].

#### Procedures

According to Shek et al.[11], the procedures of a qualitative research should be clearly presented (Principle 3). As such, the procedures for data collection are systematically described. The telephone interviews were conducted between late-March to mid-April 2006. As the Experimental Implementation

Phase took place from January 2006 to August 2006, late-March to early-April 2006 can be regarded as midway of the implementation process. While telephone interviews have the problems of psychological distance and inability to observe the nonverbal cues of the informants, its major advantage is efficiency in collecting the data. This advantage is important because many schools might reject the idea of completing questionnaires and participating in face-to-face interviews during term time. In addition, follow-up calls could be arranged if there was a need to clarify the responses of the informants.

A self-constructed, semi-structured interview guide with seven questions was used to collect information on the program implementation process. These questions were:

1. What are the responses of the students to this program?
2. What are the experiences of the workers when they implement the program?
3. Do you think this program is beneficial to the students? If yes, what are the benefits?
4. What are the good aspects of the program?
5. Which areas of the program require improvement?
6. Have you encountered any difficulties during the program implementation process? If yes, what problems have you encountered?
7. Overall, what is your evaluation of the program?

For question 1, 2, and 7, besides inviting the informants to freely narrate their experiences, the informants were asked to indicate whether their experiences were “positive”, “negative”, or “neutral”, and give examples to illustrate their answers.

Informed consent was obtained from the informants and they participated in the study in a voluntary manner. The interviews were conducted by the research assistants of the research team who were registered social workers with substantial working experience. The telephone interviews were conducted in Cantonese and the responses of the informants were jotted down during the interviews. The handwritten notes were then transcribed and analyzed.

### Data Analyses

The data were analyzed using general qualitative analyses techniques[12]. There were three steps in the process. First, the transcribed interview materials were coded to differentiate semantically different information and group semantically close information. Relevant raw codes were developed for words, phrases, and/or sentences that form meaningful units. Second, the codes were further combined to reflect higher-order attributes, such as perceived benefit in promoting holistic development in students in different domains. Finally, except Question 6, all responses were categorized in terms of whether they were positive responses, negative responses, neutral responses, or responses that cannot be determined (i.e., “undecided” responses).

The present codes were developed after several preliminary analyses of the transcripts by the researchers. In order to ensure the reliability of the coding, both intra- and inter-rater reliability on the coding was calculated. The researcher and another research assistant who was a registered social worker not affiliated with this project recoded 20 randomly selected responses for each question (except Question 6) without knowing the original codes given at the end of the scoring process.

Shek et al.[11] highlighted the importance of being aware of one’s biases and preoccupations of the researchers (Principle 4) and to address such biases in a proper perspective (Principle 5). In this study, as the researchers were the program developers, it was likely for them to believe that the implemented program was worthy and beneficial to the participants, and they tended to look for positive evidence or overlook negative evidence. Therefore, several steps were taken to guard against the above biases. First, the researchers were conscious about their researcher role and conducted this study in a disciplined manner. Second, intra- and inter-rater reliability on the coding was carried out (Principle 6). Third, multiple research assistants were involved in the data collection process (Principle 7). Finally, the procedures involved in data collection and data interpretations were systematically documented (Principle 9).

### RESULTS

#### Responses of the Students to the Program

As shown in Table 1, a total of 18 informants (64.3%) reported that the students had positive responses towards the program, while 10 informants (35.7%) reported that the students' responses towards the program were neutral. With reference to the raw narratives, 28 responses were regarded as positive responses, two responses were coded as neutral responses, and nine responses were regarded as negative responses. The percentage of intra-rater agreement was 95% and the inter-rater agreement percentage between the rater and another research assistant was 90%.

**TABLE 1**
**Responses of the Students to the Program as Perceived by the Workers**

| Workers' Perceptions of the Responses of the Students to the Program | Total Count (%) |
|---|---|
| **Overall Positive Evaluation** | 18 (64.3%) |
| *Related Positive Responses (N = 28; see examples below)* | |
| • Interactive format is attractive to students (e.g., "liked the games in the units", "liked the interactive curriculum", "liked the activity-based curriculum", "liked the flexible teaching format") | |
| • High student involvement (e.g., "active", "involved", "eager to join the program", "willing to participate") | |
| • Welcomed by students | |
| • Other responses (e.g., "relaxed", "happy", "playful", "helpful") | |
| **Overall Neutral Evaluation** | 10 (35.7%) |
| *Related Neutral Responses (N = 2; see examples below)* | |
| • "The Tier 1 Program is partially welcomed by students" | |
| • "The classes with disciplinary problems partially accept the program" | |
| **Overall Negative Evaluation** | 0 (0%) |
| *Related Negative Responses (N = 9; see examples below)* | |
| • Feeling bored | |
| • Having difficulties in discussion | |
| • Other responses (e.g., "poor reaction as the program was held after school", "poor discipline", "passive") | |
| **Total (%)** | **28 (100%)** |

#### Workers' Experiences about the Program Implementation

As shown in Table 2, 14 informants (50%) reported having positive experiences about the program implementation, nine informants (32.1%) reported having neutral feelings (i.e., a mixture of both positive and negative feelings), and five informants (17.9%) reported having negative feelings during the program implementation. Among the raw descriptions, there were 21 responses coded as positive responses, nine responses coded as neutral responses, and 20 responses coded as negative responses. Both the intra- and inter-rater agreement percentages calculated were 100%.

**TABLE 2**
**Workers' Experiences about the Program Implementation**

| Workers' Experiences about the Program Implementation | Total Count (%) |
|---|---|
| **Overall Positive Evaluation** | 14 (50%) |
| *Related Positive Responses (N = 21; see examples below)* | |
| • Happy | |
| • Believe the program is helpful to the students | |
| • Appreciate the curriculum (e.g., "rich curriculum, including wide content, interesting and various teaching methods"; "comprehensive teaching materials") | |
| • Other responses (e.g., "it is meaningful, and I like students' sharing and enjoy participating in the students' growing process"; "can have a deeper understanding on and more interaction with students"; "smooth implementation") | |
| **Overall Neutral Evaluation** | 9 (32.1%) |
| *Related Neutral Responses (N = 9; see examples below)* | |
| • "It is worthwhile and happy, but the implementation process is tight and confused" | |
| • "It is helpful to the students, but it is busy" | |
| **Overall Negative Evaluation** | 5 (17.9%) |
| *Related Negative Responses (N = 20; see examples below)* | |
| • Time constraints (e.g., "time is tight for running too many activities in each unit", "time is pressing from the confirmation to implementation") | |
| • Activity design (e.g., "have too many student worksheets") | |
| • Administrative difficulties (e.g., "the original administrative arrangement is affected", "have difficulties in coordination") | |
| • Feeling uneasy and busy (e.g., "feeling hard", "it is difficult to control class discipline", "being busy and confused", "being exhausted") | |
| **Total (%)** | **28 (100%)** |

### Perceived Benefits of the Program to the Students

As shown in Table 3, there were 34 raw descriptions on the perceived benefits of the program to the students. Except for one response that was coded as "undecided" (2.9%), the rest of the responses were coded as positive responses. These included seven responses (20.6%) indicating that the program could facilitate the holistic development of the students; five responses (14.7%) indicating that the program could build up students' peer relationships; 10 responses (29.4%) indicating that the program could strengthen students' behavioral, social, and cognitive competence; three responses (8.8%) indicating that the program could enhance students' moral values; and eight responses (23.5%) indicating that there were other benefits. Both the intra- and inter-rater agreement percentages calculated were 100%.

### Positive Aspects of the Program and Areas that Require Improvement

There were 36 raw descriptions regarding the good aspects in the project (Table 4). Except for one response (2.7%) that was coded "undecided", there were 19 responses (52.8%) indicating that the curriculum content was good, four responses (11.1%) indicating that the philosophy underlying the project was comprehensive, six responses (16.7%) indicating that activities were well designed, and six responses (16.7%) indicating that training for workers, evaluation on the project, and support to teachers were sufficient. On the other hand, there were 36 raw descriptions on the areas requiring improvement in

**TABLE 3**
**Perceived Benefits of the Program**

| **Workers' Perceptions on the Benefits of the Program** | **Total Count (%)** |
|---|---|
| **Undecided** | 1 (2.9%) |
| **Facilitating the Holistic Development of the Students** (see examples below) | 7 (20.6%) |
| • "The design matches students' developmental needs" | |
| • "It has comprehensive content that students need to know" | |
| • "It facilitates personal development" | |
| • "It enables comprehensive discussion on students' growth issues" | |
| **Building Up Peer Relationships** (see examples below) | 5 (14.7%) |
| • "It raises team spirit" | |
| • "Students can make more friends" | |
| • "Students get closer with their classmates" | |
| • "It enables group collaboration" | |
| **Strengthening Students' Behavioral, Social, and Cognitive Competencies** (see examples below) | 10 (29.4%) |
| • "There are apparent and positive changes in students' conduct" | |
| • "It enables students to discuss and reflect on daily life issues" | |
| • "It enables decision-making skills" | |
| • "It improves social skills" | |
| **Enhancing Students' Moral Values** (see examples below) | 3 (8.8%) |
| • "It transmits positive life values and helps students to grow" | |
| • "It gives students insight and enables them to build up a positive value system" | |
| **Other Responses, Such As Facilitating Students' Learning** (see examples below) | 8 (23.5%) |
| • "The materials are good and help students to learn" | |
| • "Teaching materials are systematic, and let teachers and students come across the content that has been neglected" | |
| **Total (%)** | **34 (100%)** |

the project (Table 5). Except for two responses (5.6%) indicated "none", there were 18 responses (50%) indicating that the activity design in the curriculum needed to be modified, seven responses (19.4%) suggested having sufficient time for implementation, and nine responses (25%) suggested other areas requiring improvement. The intra- and inter-rater agreement percentages calculated were 95% and 85%, respectively.

### Difficulties Encountered During Program Implementation

As shown in Table 6, there were 44 raw descriptions on the difficulties encountered by the workers during program implementation. Among them, 13 responses (29.5%) were related to difficulties in teaching and coordination, 17 responses (38.6%) were related to difficulties in time management, seven responses

(15.9%) were related to difficulties in manipulating the activity design, and seven responses (15.9%) were concerned about difficulties in handling students' responses. No intra- and inter-rater agreement percentages were calculated for the related responses.

**TABLE 4**
**Positive Aspects of the Project**

| Workers' Perceptions on the Positive Aspects of the Project | Total Count (%) |
|---|---|
| **Undecided** | 1 (2.7%) |
| **Curriculum Content , Including Teaching Manuals and Resources** (see examples below) | 19 (52.8%) |
| • "The content is rich" | |
| • "It has a clear content" | |
| • "The curriculum has a comprehensive coverage" | |
| • "It has ready-made teaching manuals" | |
| • "The instructions are detailed" | |
| • "It has sufficient resources" | |
| **Philosophy** (see examples below) | 4 (11.1%) |
| • "The philosophy behind the program is good" | |
| • "It has comprehensive rationales" | |
| • "The spirit of positive growth is agreeable" | |
| **Activity Design** (see examples below) | 6 (16.7%) |
| • "Some activities are good" | |
| • "The topics match the students' needs" | |
| • "Most of the time, there is no definite answer in the unit, and thus providing space for students to reflect" | |
| **Other Responses, Including Training and Evaluation** (see examples below) | 6 (16.7%) |
| • "It is great that both teachers and social workers can have training" | |
| • "The training is good" | |
| • "Evaluation on the project is comprehensive" | |
| • "Support to teachers is very sufficient" | |
| **Total (%)** | **36 (100%)** |

### Global Evaluation of the Program

As shown in Table 7, the responses of 22 informants (78.6%) could be regarded as positive evaluation and responses of three informants (10.7%) were regarded as neutral evaluation. Besides, two responses (7.1%) were coded as negative evaluation and one response (3.6%) was regarded as "undecided". Among the raw descriptions, there were 25 responses coded as "positive", one response coded as "neutral", and two responses coded as "negative". Both the intra- and inter-rater agreement percentages calculated were 95%.

## DISCUSSION

The primary purpose of this paper was to report interim evaluation findings on the implementation of the Tier 1 Program of the Project P.A.T.H.S. in the Experimental Implementation Phase in the academic year of 2005–06. Several phenomena could be highlighted from the findings. First, the informants felt that most of the students had positive responses to the program and half of the workers had positive experiences about the program (plus another 32.1% of the workers who had both positive and negative feelings). Second, a majority of the informants regarded the program as beneficial to the students. Third, a majority of the informants' global evaluation of the program was positive. Fourth, while the informants

**TABLE 5**
**Aspects of the Program that Require Improvement**

| Workers' Perceptions on the Aspects of the Program that Require Improvement | Total Count (%) |
|---|---|
| **None** | 2 (5.6%) |
| **Activity Design, Including Aims, Content, and Teaching Aids** (see examples below) | 18 (50%) |
| • "The aims of some units are too difficult to Secondary 1 students" | |
| • "There are too many aims in each unit" | |
| • "The number of themes can be reduced" | |
| • "The content is very broad, and not in depth" | |
| • "There is a need to modify the content to match students' needs" | |
| • "The design of some units is similar" | |
| • "There is insufficient flexibility in the program design" | |
| • "There is a need to have various teaching aids to cater for different students' needs" | |
| • "There are too many worksheets" | |
| • "The Growth Puzzle is too big" | |
| • "The reflective questions in Growth Puzzle are repeating" | |
| **Implementation Time** (see examples below) | 7 (19.4%) |
| • "It is better to have sufficient time for running the units" | |
| • "The run-down of the project is rush, and thus it needs adjustment and time plan" | |
| **Other Responses, Including the Language of the Curriculum and Administrative Issues** (see examples below) | 9 (25%) |
| • "There is a need to have an English version of the curriculum" | |
| • "It is suggested to distribute the curriculum earlier, so that the school and teachers can make appropriate adjustment" | |
| • "It is suggested to have more sharing among schools in order to understand the implementation and effectiveness of the program in different schools" | |
| • "There is a need to solve the manpower issues" | |
| • "There is a need to reduce teachers' heavy workload" | |
| **Total (%)** | **36 (100%)** |

appreciated the program, they also identified problems in the implementation process and proposed suggestions for refinement. Taken as a whole, these findings reinforced the findings arising from other evaluation studies that the program was helpful to the students, and both the students and workers had positive perceptions of the program[7,8,9].

Regarding the negative experiences expressed by the workers and the problems they encountered during the process, there are four factors contributing to these observations. First, as implementation of positive youth development program utilizing the curricula approach is relatively new in Hong Kong, teachers need time to adjust to the mode of teaching (e.g., using games and interactive teaching methods) that is different from the traditional didactic form of teaching. Second, as it is not common for social workers and teachers to use teaching manuals to deliver a program, they might find the program inflexible. Third, as the implementation period was rather short (January 2006 to August 2006), the expressed concern about time management difficulty is expected. Finally, as the program was conducted by social workers in collaboration with the teachers, they might need time to make the collaboration more streamlined.

**TABLE 6**
**Difficulties Encountered in Program Implementation**

| Workers' Perceptions on the Difficulties Encountered in Program Implementation | Total Count (%) |
|---|---|
| **Teaching and Coordination** (see examples below) | 13 (29.5%) |
| • "Not all the teachers are interested in running the activities" | |
| • "Social workers may not get acquainted with the students, and it is difficult to handle class discipline" | |
| • "The number of students per class is too high" | |
| • "Teacher's role is not clear" | |
| • "Division of labor is not clear" | |
| • "Teachers feel uneasy to collaborate with social workers" | |
| • "Teachers have to spend much time and energy to prepare the activities" | |
| • "There is insufficient flexibility in arranging manpower and time for the project" | |
| **Time Management** (see examples below) | 17 (38.6%) |
| • "The implementation time of the project is short" | |
| • "There is a need to coordinate different parties in order to spare sufficient time to finish all the content in the curriculum" | |
| • "Time is limited for running units" | |
| • "It is difficult to control time" | |
| **Activity Design** (see examples below) | 7 (15.9%) |
| • "It is too academically based, and there is a difficulty in matching the activity design" | |
| • "The curriculum design is not very smooth" | |
| • "Tier 1 Program lacks flexibility" | |
| • "Some activities are repeating" | |
| **Students' Responses** (see examples below) | 7 (15.9%) |
| • "Students have low motivation to participate" | |
| • "There is low student involvement" | |
| • "It is difficult to handle poor class discipline" | |
| **Total (%)** | **44 (100%)** |

Shek et al.[11] suggested that it is important to consider alternative explanations in the interpretations of qualitative evaluation findings (Principle 10). There are several possible alternative explanations for the present findings. First, the findings can be explained in terms of demand characteristics. However, this explanation is not likely because the informants were encouraged to voice their views without restriction and negative voices were in fact heard. In addition, as telephone interviews were conducted (i.e., no face-to-face interaction), the tendency for the informants to express negative views would be higher. The second alternative explanation is that the findings were due to selection bias. However, this argument is not strong as the schools and NGOs that provided school social work services were randomly selected. The third alternative explanation is that the positive findings were due to ideological biases of the researchers. As several safeguards were used to reduce biases in the data collection and analysis processes, this possibility is not high.

With reference to Principle 12 of Shek et al.[11], there are several limitations of the study. First, it should be noted that in order to gain efficiency, the price one has to pay in telephone interviews is the inability to collect in-depth information. Nevertheless, because schools are always reluctant to participate in in-depth studies during term time, telephone interviews can be regarded as a pragmatic way of collecting data. Second, although other mechanisms of evaluation have been carried out[7,8,9], the

**TABLE 7**
**Global Evaluation of the Program**

| **Workers' Global Evaluation of the Program** | **Total Count (%)** |
|---|---|
| **Undecided** | 1 (3.6%) |
| **Overall Positive Evaluation** | 22 (78.6%) |
| *Related Positive Responses (N = 25; see examples below)* | |
| • Helpful to students (e.g., "it is beneficial to students' growth", "it can enhance students' skills and competence") | |
| • Resources for students (e.g., "there is extra resources for student activity", "the program has sufficient resources") | |
| • Curriculum design (e.g., "it has a systematic curriculum design", "the topics covered in the curriculum are broad", "it meets students' needs", "the teaching package is comprehensive", "it provides progressive personal growth curricula for Secondary 1 to 3 students", "it has sufficient flexibility, and is easy to adapt") | |
| • Other responses (e.g., "it has sufficient support to teachers", "it raises the sense of belonging in the class", "it enables teachers to discover students' qualities in other domains besides academic performance", "it has training for teachers and enables them to acquire more knowledge") | |
| **Overall Neutral Evaluation** | 3 (10.7%) |
| *Related Neutral Responses (N = 1)* | |
| • "It is suggested to employ other teachers to teach students, so as to reduce in-service teachers' workload" | |
| **Overall Negative Evaluation** | 2 (7.1%) |
| *Related Negative Responses (N = 2)* | |
| • "The project is ambitious in term of time and teaching" | |
| • "Teachers have done a lot, and it is doubtful whether the benefit will be proportional to the sacrifice" | |
| **Total (%)** | **28 (100%)** |

inclusion of other qualitative evaluation strategies, such as in-depth individual interviews, would be helpful to further understand the subjective experiences of the program participants. Finally, although the principles proposed by Shek et al.[11] were upheld in this study, peer checking and member checking (Principle 8) were not carried out in this study because of time and manpower constraints. Furthermore, the researchers were not able to construct "thick descriptions" based on telephone interview data. Despite these limitations, this study provides pioneering interim evaluation findings supporting the positive nature of the Project P.A.T.H.S. and its effectiveness in promoting holistic youth development among Chinese adolescents in Hong Kong.

### ACKNOWLEDGMENTS

The preparation for this paper and the Project P.A.T.H.S. were financially supported by the Hong Kong Jockey Club Charities Trust.
